# Dominance of African racial ancestry in honey bee colonies of Mexico 30 years after the migration of hybrids from South America

**DOI:** 10.1111/eva.13738

**Published:** 2024-06-24

**Authors:** María de Jesús Aguilar‐Aguilar, Jorge Lobo, E. Jacob Cristóbal‐Pérez, Francisco J. Balvino‐Olvera, Gloria Ruiz‐Guzmán, José Javier G. Quezada‐Euán, Mauricio Quesada

**Affiliations:** ^1^ Laboratorio Nacional de Análisis y Síntesis Ecológica Escuela Nacional de Estudios Superiores, Unidad Morelia Morelia Michoacán Mexico; ^2^ Posgrado en Ciencias Biológicas, Unidad de Posgrado, Edificio D, 1° Piso, Circuito de Posgrados Ciudad Universitaria Coyoacán Mexico; ^3^ Laboratorio Binacional UNAM‐UCR Universidad Nacional Autónoma de México Morelia Michoacán Mexico; ^4^ Escuela de Biología Universidad de Costa Rica San Pedro Costa Rica; ^5^ Departamento de Apicultura Tropical, Campus de Ciencias Biológicas y Agropecuarias Universidad Autónoma de Yucatán Mérida Mexico; ^6^ Instituto de Investigaciones en Ecosistemas y Sustentabilidad, Universidad Nacional Autónoma de México Morelia Michoacán Mexico

**Keywords:** Africanized honey bees, *Apis mellifera*, managed and feral honey bees, mitochondrial DNA, nuclear DNA, racial ancestry

## Abstract

The Africanized honey bee, a hybrid of *Apis mellifera scutellata* from Africa with European subspecies, has been considered an invasive species and a problem for beekeeping. Africanized bees arrived in Mexico in 1986, 30 years after their accidental release in Brazil. Although government programs were implemented for its eradication, Africanized populations persist in Mexico, but precise information on the patterns of genetic introgression and racial ancestry is scarce. We determined maternal and parental racial ancestry of managed and feral honey bees across the five beekeeping regions of Mexico, using mitochondrial (mtDNA, COI‐COII intergenic region) and nuclear markers (94 ancestrally informative SNPs), to assess the relationship between beekeeping management, beekeeping region, altitude, and latitude with the distribution of maternal and parental racial ancestry. Results revealed a predominantly African ancestry in the Mexican honey bees, but the proportion varied according to management, beekeeping regions, and latitude. The Mexican honey bees showed 31 haplotypes of four evolutionary lineages (*A*, *M*, *C*, and *O*). Managed honey bees had mitochondrial and nuclear higher proportions of European ancestry than feral honey bees, which had a higher proportion of African ancestry. Beekeeping regions of lower latitudes had higher proportions of African nuclear ancestry. Managed and feral honey bees showed differences in the proportion of maternal and nuclear racial ancestry. Managed honey bees from the Yucatan Peninsula and feral honey bees had a higher mtDNA than nuclear proportions of African ancestry. Managed honey bees, except those on the Yucatan Peninsula, had a higher nuclear than mtDNA proportion of African ancestry. Our study demonstrates that Africanized honey bee populations are genetically diverse and well established in Mexico, which highlights the limitations of management and government programs to contain the Africanization process and demands the incorporation of this lineage in any breeding program for sustainable beekeeping.

## 1 INTRODUCTION

Biological invasions occur when species establish outside their natural distribution, spread, and ecologically alter the invaded community. Despite their negative effects on natural ecosystems, invasive species may serve as models to study the ecological and genetic factors associated with adaptation to new environments (Lucek et al., 2012; Rius & Darling, 2014). As invasive species expand from their original distribution, they may hybridize with local species, varieties, or subspecies (Pfennig et al., 2016). The positive or negative effects of the genetic admixture of invasive species with other subspecies or native species is a debated topic that requires further genetic and ecological studies on the consequences of hybridization (Rius & Darling, 2014). The geographic variation of hybridization and introgression and their role in the adaptive process to natural or anthropogenic conditions have helped understand the importance of genetic admixture in biological invasions (Calfee et al., 2020; De Carvalho et al., 2010; Fitzpatrick et al., 2010).

The expansion of Africanized honey bees throughout the Americas is among the most extensive bee invasions in the world (Scott Schneider et al., 2004; Winston, 1992) and represents a good model for understanding the patterns of genetic admixture with other subspecies (e.g. European honey bees). One of the main factors affecting the ability of a species to invade and spread in new environments is the mating system (Sakai et al., 2001). In the honey bee, the mating system relies on the aggregation of thousands of males from many colonies at drone congregation areas (DCAs), where virgin honey bee queens visit to mate with tens of drones (Baudry et al., 1998; Jaffé et al., 2009; Koeniger & Koeniger, 2000). In these mating congregation areas, gene flow between neighboring honey bee subspecies is common and introgression can proceed quickly (Jensen et al., 2005; Scott Schneider et al., 2004).

*Apis mellifera* L. comprises 33 subspecies (Ilyasov et al., 2020), grouped into at least five lineages, namely the African (*A*), Western European (*M*), Eastern European (*C*), Asian (*O*), and Arabian Peninsula and African Horn (*Y*) (Arias & Sheppard, 1996; Cridland et al., 2017; Garnery et al., 1993; Ruttner, 1988; Wallberg et al., 2014; Whitfield et al., 2006). Genetic differences between subspecies are the result of adaptation to a wide range of environments and geographical isolation, and are expressed as high variation in phenotypic traits, such as differences in morphology, behavior, and disease susceptibility (Collins et al., 1982; Dietemann et al., 2009; Harpur et al., 2020; Wallberg et al., 2014). African subspecies are adapted to warm climates, tend to swarm more frequently, and express higher levels of defensive behavior (Harpur et al., 2020; Ruttner, 1988). European subspecies (e.g. *A. mellifera mellifera* L.) are adapted to temperate climates, store larger quantities of honey, and have a more docile behavior than other subspecies (Ruttner, 1988).

In the America the first introductions of *A. mellifera* from Europe in the 1600s presumably only included the lineages *C* and *M* (Clarke et al., 2001; Whitfield et al., 2006). More recently, in the 1950s, honey bee colonies of the *A. mellifera scutellata* L. subspecies were imported into Brazil and bred with European subspecies under controlled conditions to improve honey production under neotropical environmental conditions (Kerr, 1957). However, colonies of *A. m. scutellata* escaped and interbred with locally managed honey bees of European origin, producing a hybrid hereafter called Africanized honey bee (Winston, 1992). Since 1957, the spread of the Africanized honey bees from Brazil through South and Central America occurred at an estimated rate of 100–300 km per year (Taylor, 1977), being detected in Mexico for the first time near the border with Guatemala in 1986 (Fierro et al., 1987). According to genetic evidence, the geographical distribution of Africanized bee populations is limited mainly by climatic barriers in the northern and southern hemispheres (e.g., Calfee et al., 2020; Porrini et al., 2020).

In response to the great concern associated with the apparent difficulties in managing Africanized honey bees, principally with their highly defensive behavior, the governments of several countries developed programs that aimed to slow down the Africanized honey bees' progress (Quezada‐Euán, 2007). In anticipation of their arrival to Mexico, in 1984, the government of this country developed an official policy to attempt to control the expansion of Africanized honey bees and their hybridization with European honey bees already established in managed apiaries (Programa Nacional para el Control de la Abeja Africana ‐PNCAA). Together, the United States and Mexico governments developed a program that aimed to massively capture Africanized honey bees, hanging thousands of cardboard boxes in orchards and forests across a broad belt of Mexico, from the Pacific to the Gulf, between 1986 and 1987 (Booth, 1988). Even so, by 1993, Africanized honey bees had been detected throughout the country (Quezada‐Euán, 2007).

Several methods have been used to study the Africanization process, which includes morphometry and maternal (mitochondrial DNA) and parental genetic markers (isozymes, microsatellites, and recently SNPs and whole genome sequencing). The information obtained with these techniques has allowed the identification of gene flow between European and Africanized honey bees during and after the Africanization process. As the Africanized honey bees moved from south to north, they hybridized with the European honey bees already established in each country. Studies about the Africanization of honey bees in the Americas have found geographic variation in the proportion of Africanization, which has been related to several factors. In Brazil, Colombia, and Nicaragua, through genetic analysis, it was found that the proportion of Africanized honey bee colonies was associated with altitudinal gradients and the variation in climatic conditions (Diniz et al., 2003; Düttmann et al., 2022; Prada et al., 2009; Quezada‐Euán et al., 2003; Tibatá et al., 2018). Studies conducted in Brazil, Costa Rica, and Mexico, using morphometrics, isozymes, and mitochondrial DNA (mtDNA), have revealed that the frequency of African markers in honey bee colonies was negatively associated with the density of managed European colonies before “Africanization” (Lobo et al., 1989; Moritz & Meusel, 1992; Quezada‐Euáun, 2000). Another factor that has influenced the levels of genetic admixture has been the continuous introduction of European honey bee queens into apiaries as part of “genetic improvement” programs towards more docile and productive bees (Kraus et al., 2007). An example of this kind of program was the importation and release of thousands of European queen bees during the initial expansion of Africanized honey bees in Brazil (Kerr, 1967). In Mexico, government agencies from 1984 until now have promoted the distribution of European bee queens to beekeepers as part of a national genetic management program of honey bees (Programa Nacional para el Control de la Abeja Africana ‐PNCAA‐2010). Due to the repetitive introduction of queen bees or as a product of beekeeping management practices, racial ancestry may differ between apiaries and feral Africanized honey bees, especially in localities with more developed and modern beekeeping enterprises.

Previous studies in Mexico, one of the countries with the highest concentration of managed honey bee colonies worldwide (Quezada‐Euán, 2007), performed at a regional scale have documented the Africanization process principally in managed colonies from Southern Mexico (Clarke et al., 2002; Kraus et al., 2007; Quezada‐Euan & Jesus May‐Itza, 2001; Quezada‐Euán & Medina, 1998; Quezada‐Euáun, 2000) and to a lesser extent in the north of the country (Medina‐Flores et al., 2015; Silva‐Contreras et al., 2019; Zamora et al., 2008). Domínguez‐Ayala et al. (2016) analyzed the racial admixture (based on morphometrics and PCR‐RFLP of COI‐COII region) of managed honey bee colonies from several states in Mexico, showing that colonies descended in similar proportions from African and European lineages and that the degree of Africanization is likely climate‐driven. However, the genetic marker used in this study (mitochondrial DNA) could only trace the maternal origin of colonies. There is a need for information about the patterns of introgression at nuclear loci, which allows elucidating more clearly the levels of racial admixture, as well as an analysis of racial ancestry of feral honey bee populations across México.

In this study, we determined maternal and parental racial ancestry in managed and feral honey bee colonies in the five beekeeping regions of Mexico using sequence data from the COI‐COII mtDNA intergenic region and 94 single nucleotide polymorphisms (SNPs), which are ancestrally informative (Chapman et al., 2015). We assess (1) in detail the degree of Africanization 30 years after the first report in the country and (2) the relationship between beekeeping management, beekeeping region, altitude, and latitude with the distribution of COI‐COII haplotypes and the proportion of African nuclear ancestry. This analysis is a unique opportunity to more clearly understand the migration process of the invasive Africanized honey bees almost 60 years after it started from Brazil and the genetic capabilities of an insect species to adapt to different biomes across Mexico and to hybridize and maintain gene flow between feral and managed conspecifics.

## 2 MATERIALS AND METHODS

### 2.1 Study regions and colonies sampling

**FIGURE 1:**
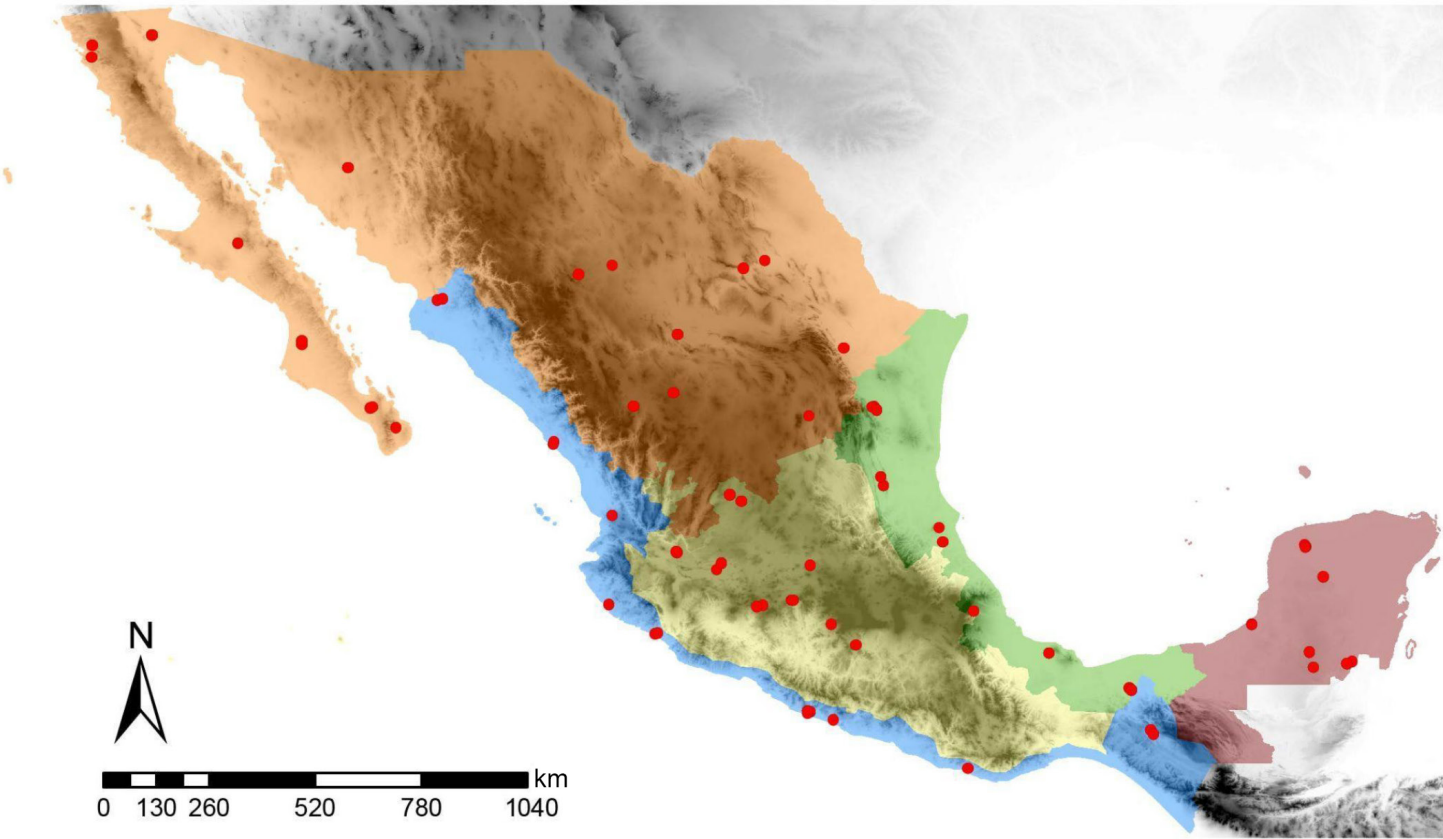
Map showing the location of sampled apiaries and the five beekeeping regions of Mexico: North in orange (*n* = 130; 80 managed and 50 feral colonies), Central Highlands in yellow (*n* = 76; 49 managed and 27 feral colonies), Pacific Coast in blue (*n* = 108; 83 managed and 25 feral colonies), Gulf Coast in green (*n* = 110; 89 managed and 21 feral colonies), and the Yucatan Peninsula in red (*n* = 85; 62 managed and 23 feral colonies). The map was generated using ArcMap 10.8.2.

In Mexico, the regionalization of beekeeping activities consists of five beekeeping regions: North, Central Highlands, Pacific Coast, Gulf Coast, and the Yucatan Peninsula; which are different in climatic conditions (e.g., precipitation, temperature) (Domínguez‐Ayala et al., 2016; Labougle & Zozaya, 1986). Our study was conducted in these beekeeping regions, with the assistance of the Beekeeping Product System Association of Mexico, between 2017 and 2019. In each beekeeping region, we collected 10–16 apiaries (Figure 1). We collected 3–12 honey bee colonies per apiary of managed bees and from 1 to 10 feral colonies (Table S1). We collected twenty worker bees per colony from managed and feral honey bee colonies and preserved them in 90% ethanol at −20°C until DNA extraction. Managed honey bee colonies consisted of beehives under beekeeping management practices, such as systematic honey bee queen replacement, disease control, and supplemental feeding. Feral honey bee colonies were those found in natural cavities or human constructions near the apiary or swarms captured with swarm traps (Coulson et al., 2005; Schiff et al., 1994). All feral colonies were located within a 5 km radius of the apiary. All apiaries were georeferenced to analyze the correlation between honey bee racial ancestry with latitude and altitude. Selection and manipulation of the honey bee colonies were carried out with the assistance of the beekeepers. In total, 509 honey bee colonies were collected from 64 apiaries, comprising 363 managed and 146 feral colonies throughout Mexico.

### 2.2 DNA extraction

We extracted total DNA from bee legs using a modified Cetyltrimethylammonium Bromide (CTAB) protocol (Doyle & Doyle, 1987). For mtDNA analysis, we analyzed a single honey bee worker bee per colony because, due to its maternal inheritance, only a single worker bee is needed to determine the maternal ancestry of the colony. For nuclear ancestry analysis, we pooled DNA from 20 worker bees per colony, ensuring a more comprehensive representation of the nuclear racial ancestry of the colony's patrilines. We chose this approach based on the strong correlation between the allele frequencies obtained from DNA samples extracted and genotyped in a pool (pooled DNA extracted from 20 worker bees per colony) regarding the DNA extracted and genotyped individually (DNA extracted from 20 worker bees separately) (*r* = 0.999; Figure S1). Analyzing the pooled DNA of worker bees allowed us to increase the sample size per colony while optimizing the cost of DNA sequencing.

### 2.3 Mitochondrial DNA analysis

Of the 509 honey bee colonies collected, 495 were analyzed using the highly polymorphic COI‐COII intergenic region. This region was amplified by standard PCR using the primers E2 (5′–GGCAGAATAAGTGCATTG–3′) and H2 (5′–CAATATCATTGATGACC–3′) as described by Garnery et al. (1993), with some modifications. The amplified PCR products were sent to Macrogen Inc. (Seoul, Korea) for direct Sanger sequencing in both directions. Sequences were manually checked for base calling and aligned, using the Geneious software (v11.0.5; Biomatters), with reference sequences of the five described *A. mellifera* lineages: African (*A*), Western European (*M*), Eastern European (*C*), Asian (*O*), and Arabian Peninsula and African Horn (*Y*) (Arias & Sheppard, 1996; Cridland et al., 2017; Garnery et al., 1993; Ruttner, 1988; Wallberg et al., 2014; Whitfield et al., 2006), published in the GenBank (http://www.ncbi.nlm.nih.gov). All haplotype sequences obtained were submitted to the GenBank NCBI database (Table S2). To determine the haplotype lineages, we constructed a phylogenetic tree using the maximum likelihood method (ML), with a bootstrap value of 1000 replicates using MEGA 10.2 (Kumar et al., 2018). Tree representation was prepared using the software iTOL v.5.5.1 and Adobe Illustrator v.24.2.

The new haplotype variants were numbered based on the number of the last variant in each haplotype, according to the nomenclature system of Chávez‐Galarza et al. (2017). To illustrate the haplotype frequencies and relationships, a haplotype network was constructed based on statistical parsimony (Templeton et al., 1992) using the haploNet function of the *pegas* package in R (Paradis, 2010). The graphic representation of the haplotype network was prepared using the software Adobe Illustrator v. 24.2. Mitochondrial Africanization was calculated as the percentage of African haplotypes present in each apiary.

### 2.4 Analysis of racial admixture at SNPs

Of the 509 honey bee colonies collected, 501 were genotyped at 94 ancestrally informative nuclear SNPs (Chapman et al., 2015). These SNPs distinguish between the three lineages most abundant in the Americas: *C*, *M*, and *A* (Chapman et al., 2015). Lineages *O* and *Y* have not been detected in the Americas, except for a few reports of *O* (Magnus & Szalanski, 2010; Zárate et al., 2022). The amplicons containing the SNPs were amplified in four PCR multiplex sets following the authors' recommendations and then sequenced using the MiniSeq platform (Illumina, San Diego, CA, USA) (Chapman et al., 2015). Reads generated from sequencing were trimmed and filtered for quality and length using Trimmomatic software (Bolger et al., 2014). Subsequently, filtered reads for each colony were aligned to reference genome AMEL_v4.5 (Elsik et al., 2014) using BWA v0.7.5 (Li & Durbin, 2010). The frequencies of each allele variant were obtained using the program VARSCAN v2.3.7 (Koboldt et al., 2009).

In our study, the racial admixture of each colony was estimated using the classical formula of racial admixture (Lobo et al., 1989; Lobo & Krieger, 1992):

(1) $$p_{\mathrm{ij}}=Ap_{\mathrm{Aij}}+Mp_{\mathrm{Mij}}+Cp_{\mathrm{Cij}}$$

where $p_{\mathrm{ij}}$ is the frequency of allele *i* of the *j* locus, *A* is the admixture proportion of the *A* lineage; $p_{\mathrm{Aij}}$ is the frequency of this allele in the *A* lineage; *M* is the admixture proportion of the *M* lineage; $p_{\mathrm{Mij}}$ is the frequency of this allele in the *M* lineage; *C* is the admixture proportion of the *C* lineage; $p_{\mathrm{Cij}}$ is the frequency of this allele in the *C* lineage. The maximum likelihood estimation of *A*, *M*, and *C* was obtained by considering that the probability of observing the frequency ${\hat{p}}_{\mathrm{ijk}}$ of the allele i at the SNP *j* in the colony *k* and can be defined using a binomial sampling distribution, where $r_{\mathrm{ijk}}$ is the number of copies of that allele in the sample of 40 alleles (20 workers, $r_{\mathrm{ijk}}$ calculated by ${\hat{p}}_{\mathrm{ijk}}=r_{\mathrm{ijk}}/40$). Therefore, the probability to sample $r_{\mathrm{ijk}}$ is

(2) $$\mathrm{Pr}\left(r_{\mathrm{ijk}}={\hat{p}}_{\mathrm{ijk}},p_{\mathrm{ijk}}\right)=\frac{40!}{r_{\mathrm{ijk}}!\left(40-r_{\mathrm{ijk}}\right)!}\;{p_{\mathrm{ijk}}}^{r_{\mathrm{ijk}}}{\left(1-p_{\mathrm{ijk}}\right)}^{40-r_{\mathrm{ijk}}}$$

where $p_{\mathrm{ijk}}$ is the frequency of that allele in the whole population of workers in the colony. Finally, the probability of observing a gene frequency vector for 94 SNPs in a colony *k* ${\hat{p}}_{k}$, based on the admixture proportions *A*, *M*, and *C*, is

(3) $$\mathrm{Pr}\left({\hat{p}}_{k},A,M,C\right)=\prod_{j=1}^{94}\mathrm{Pr}\left({\hat{p}}_{\mathrm{ijk}},p_{\mathrm{ijk}}\right)\;\mathrm{Pr}\left(p_{\mathrm{ijk}};A,M,C\right)$$

where $\mathrm{Pr}\left(p_{\mathrm{ijk}};A,M,C\right)$ is Equation 1. Function (3) was used as the likelihood function, and the parameters *A*, *M*, and *C* were those that minimized the negative logarithm of the function. These values were obtained using the nlm function from the base R *stats* package in R v4.2.0 (R Core Team, 2022). To visualize the admixture of each honey bee colony, we constructed a ternary plot using the *ggtern* package in R (Hamilton & Ferry, 2018).

### 2.5 Statistical analysis

We used generalized linear models using PROC GLIMMIX in SAS (SAS Institute Inc.) to test differences in the proportion of *A*, *C*, and *M* lineages, using the nuclear SNPs data, between beekeeping regions and beekeeping management. In the models, we used the proportion of each lineage (*A*, *C*, *M*) (Poisson distribution with a log link function) as the response variable. Beekeeping region, management types, and the interaction beekeeping region*management were included as fixed factors and the apiary as a random effect. We used the ilink function to calculate back‐transformed means. We adjusted *p*‐values for multiple comparisons using Tukey‐adjustments.

In our study, the *A* lineage was the most abundant and showed a negative correlation with the proportion of the *C* lineage. We used Generalized Additive Models for Location, Scale, and Shape (GAMLSS) to determine (a) the relationship of latitude and altitude with the proportion of African mitochondrial haplotypes (fitted to a binomial distribution) and (b) the relationship of latitude, altitude, and the mitochondrial lineage (African or European) with the proportion of African nuclear ancestry (fitted to GB1 distribution) for managed and feral honey bee colonies. To determine the appropriate distribution family of the data, we use the *performance* package in R (Lüdecke et al., 2021). The models included altitude, latitude, and mitochondrial lineage (African or European) as fixed factors and the apiary as a random factor. Models were built using the *gamlss* package in R (Rigby & Stasinopoulos, 2005). The best‐fitting model selection was based on the Akaike Information Criterion (AIC).

To assess the differences between mitochondrial and nuclear proportions of the three lineages in each beekeeping region, we calculated the 95% confidence intervals using the confidence interval formula for proportions: Confidence Interval (CI) = *p̂* ± z*√*p̂*(1−*p̂*)/*n*, where *p̂* is the proportion of African ancestry, *z* is the *z*‐value (*z* = 1.96 for 95% CI) and *n* is the sample size. For mitochondrial estimates, *n* is the colony number. For nuclear estimates, n is equal to 2*n + 12*n since each colony has two alleles from the queen (2*n*) plus 12 haploid alleles from the drones (12*n, which is a conservative estimated number of mating frequencies of honey bee queens). We considered overlapping 95% confidence intervals to indicate non‐significance differences. Graphs were plotted using the *ggplot* package v.3.4.2 in R (Wickham et al., 2022).

## 3 RESULTS

### 3.1 Mitochondrial analysis

**FIGURE 2:**
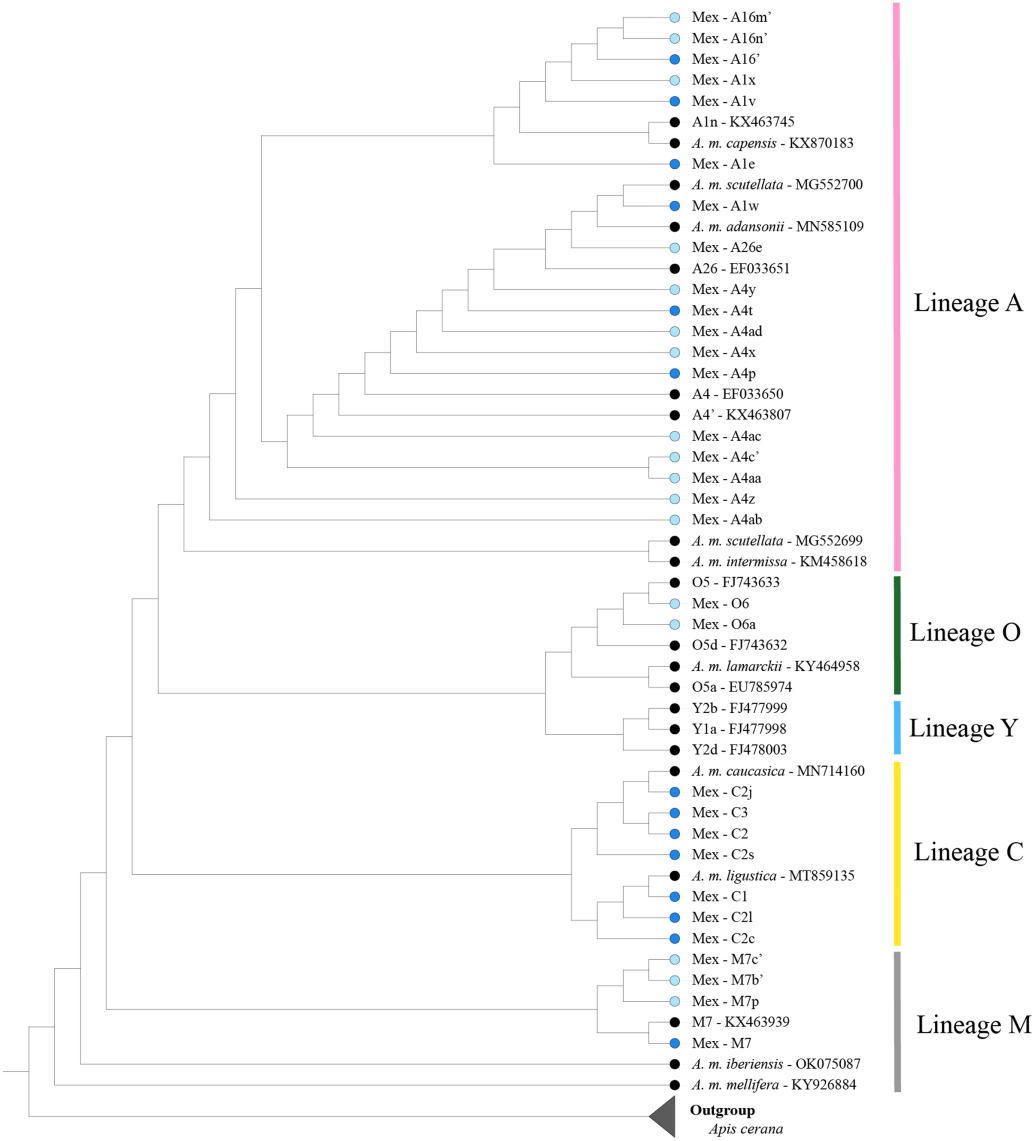
Maximum‐likelihood phylogenetic tree of haplotypes identified for honey bee populations of Mexico. Blue light circles indicate novel haplotypes, blue circles indicate haplotypes previously reported and found in Mexico, and black circles indicate reference haplotypes obtained from GenBank. Accession numbers of sequences utilized as reference are indicated in the figure.

**FIGURE 3:**
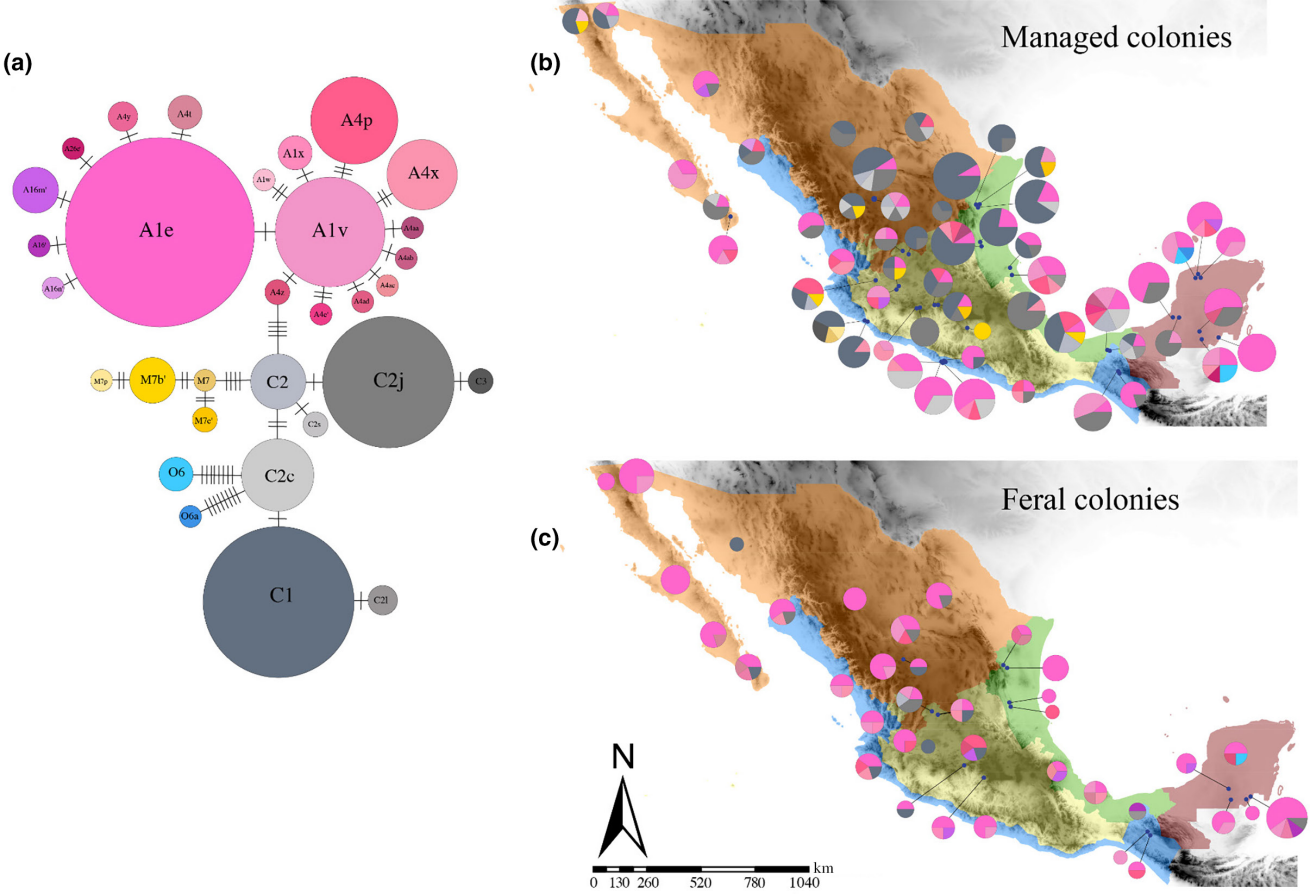
Frequencies and geographic distributions of mtDNA COI‐COII haplotypes in Mexico. (a) Haplotype network; (b, c) Geographic distribution of haplotypes in managed and feral honey bee colonies in the five beekeeping regions of Mexico: North in orange, Pacific Coast in blue, Gulf Coast in green, Central Highlands in yellow, and the Yucatan Peninsula in red. In the haplotype network and the pie charts the colors pink, gray, yellow, and blue represent haplotypes of African, Eastern European, Western European, and Asian lineages, respectively. Circles are proportional to the sample size. Maps were generated using ArcMap 10.8.2.

Mitochondrial DNA sequences revealed thirty‐one different haplotypes among the 495 samples analyzed, distributed in four evolutionary lineages as follows: eighteen haplotypes corresponded to the *A* lineage, seven to *C*, four to *M*, and two to *O* lineages. A high proportion (54%) of haplotypes were observed for the first time in Mexico (12 of the lineage *A*, three of the *M* lineage, and two of the *O* lineage) (Figure 2). The haplotype network showed four groups corresponding to four evolutionary lineages (Figure 3a). The *A* lineage (*n* = 287, 58%) (number of sequences and proportion of the total, respectively) was the most frequent, followed by the *C* lineage (*n* = 192, 38.8%), the *M* lineage (*n* = 11, 2.2%), and the *O* lineage (*n* = 5, 1%). We recorded 18 haplotypes of the *A* lineage, which varied in frequency and geographical distribution. Haplotypes A1e, A1v, A4p, and A4x were represented as follows: 35%, 8.5%, 5.1%, and 3.2%, respectively. These haplotypes were distributed in all beekeeping regions, both in managed and feral colonies, except for haplotype A4x, which was not present in the North region. The haplotypes A1w, A1x, A4t, A4y, A4z, A16′, A16m′, A16n′, A4aa, A4ab, A4ac, A4ad, A4c′, and A26e were recorded in frequencies lower than 1% (Figure 3b,c). Eastern European haplotypes were detected mainly in managed honey bee colonies. Haplotypes C1 (*n* = 93; 18.8%), C2j (*n* = 65; 13.8%), and C2c (*n* = 17; 3.4%) were the most frequent. The C2l, C2s, and C3 haplotypes were recorded in frequencies below 1%. The C2j haplotype was the only one of the *C* lineage detected in the five beekeeping regions. The western European haplotypes (*n* = 11; 2.2%) were exclusively found in managed honey bee colonies of four beekeeping regions (North, Central highlands, Pacific coast, and Gulf coast). Haplotypes of the *O* lineage (*n* = 5; 0.2%) were only detected in the Yucatan Peninsula region, mainly in managed colonies (Figure 3b,c. Table S3).

The GAMLSS analysis showed that altitude and latitude were negatively related to the proportion of African haplotypes in managed colonies but not in feral colonies (Table 1). In managed colonies, a significant negative relationship was observed between the proportion of African haplotypes with both altitude (estimate = −0.0006, SE = 0.0002, *t*‐value = −3.413, *p* = 0.0003) and latitude (estimate = −0.1061874, SE = 0.0347606, *t*‐value = −3.055, *p* = 0.00244).

**TABLE 1:** Results of the GAMLSS Models showing the relationship between altitude and latitude with the proportion of African mtDNA haplotypes of managed and feral honey bee colonies.

Management	Independent variable	Estimate	SE	*t* value	Pr(>|*t*|)
Managed colonies	Intercept	2.4652698	0.7349474	3.354	0.000894***
	Altitude	−0.0006908	0.0002024	−3.413	0.000728***
	Latitude	−0.1061874	0.0347605	−3.055	0.002447**
Feral colonies	Intercept	2.9476068	1.8808821	1.567	0.12
	Altitude	−0.0005627	0.0003496	−1.61	0.11
	Latitude	−0.0183332	0.0808666	−0.227	0.821

Asterisks indicate level of statistical significance: *p ≤ 0.05, **p ≤ 0.01, ***p ≤ 0.001.

### 3.2 Nuclear analysis based on SNPs

**FIGURE 4:**
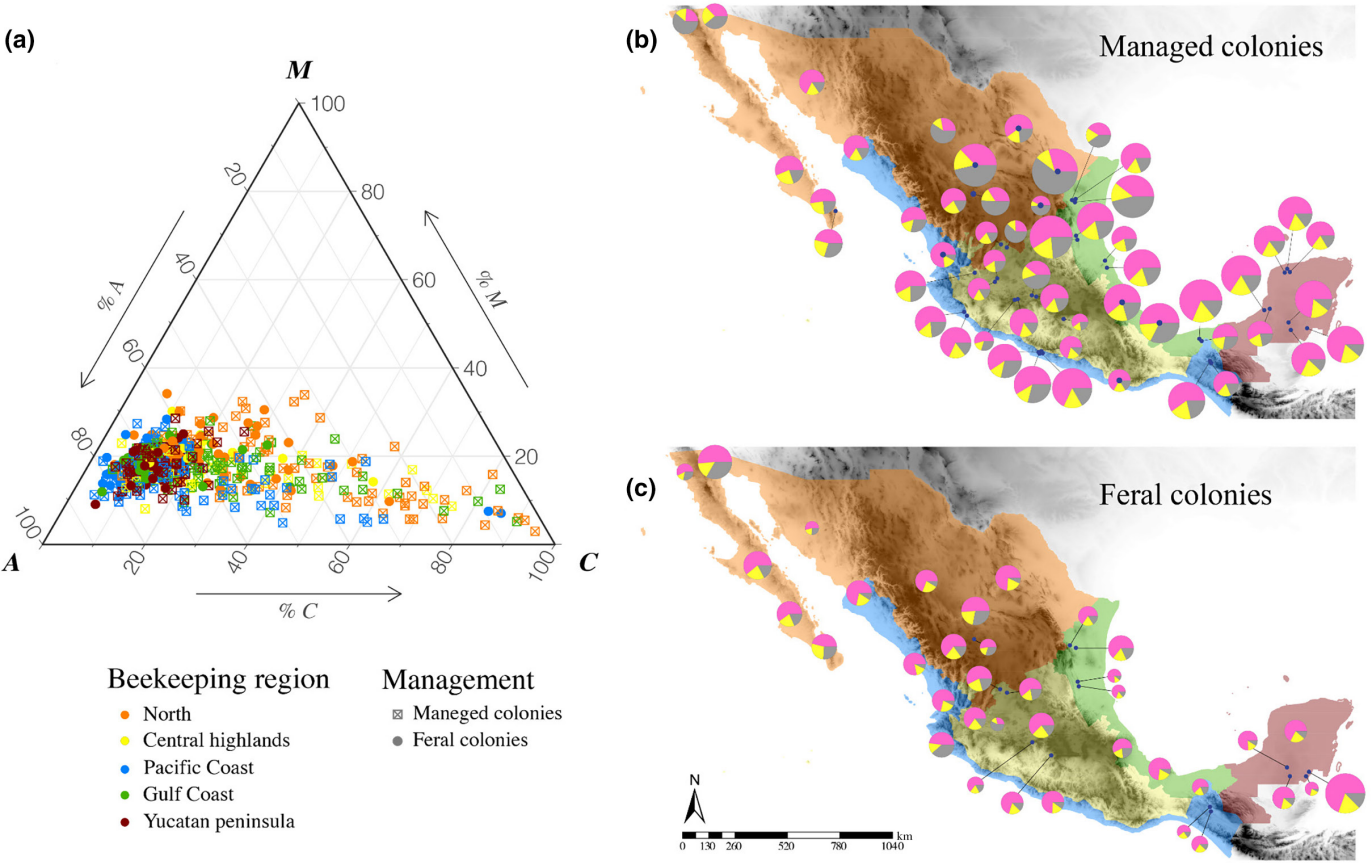
Nuclear racial Admixture in Mexican honey bee colonies. (a) Ternary plot showing the racial admixture of the three principal ancestral lineages (*A*, *C*, *M*) in honey bee colonies. Shapes indicate management (feral = circle and managed = squared plus). (b, c) Geographic distribution of nuclear racial admixture in (b) managed and (c) feral colonies in the five beekeeping regions of Mexico: North in orange, Pacific Coast in blue, Gulf Coast in green, Central Highlands in yellow, and the Yucatan Peninsula in red. Pie charts represent the average of *A* (pink), *C* (gray), and *M* (yellow) nuclear admixture in each apiary; pie size is proportional to sample size. Maps were generated using ArcMap 10.8.2.

Based on nuclear data, the 501 Mexican honey bee colonies analyzed showed that a predominant proportion of their ancestry originates from the *A* lineage (0.59), followed by *C* (0.24) and *M* lineages (0.17). Feral honey bee colonies tended to have a higher proportion of African ancestry than managed honey bee colonies, whereas managed honey bee colonies tended to have a higher proportion of *C* ancestry (Figure 4a–c; Figure S2).

**FIGURE 5:**
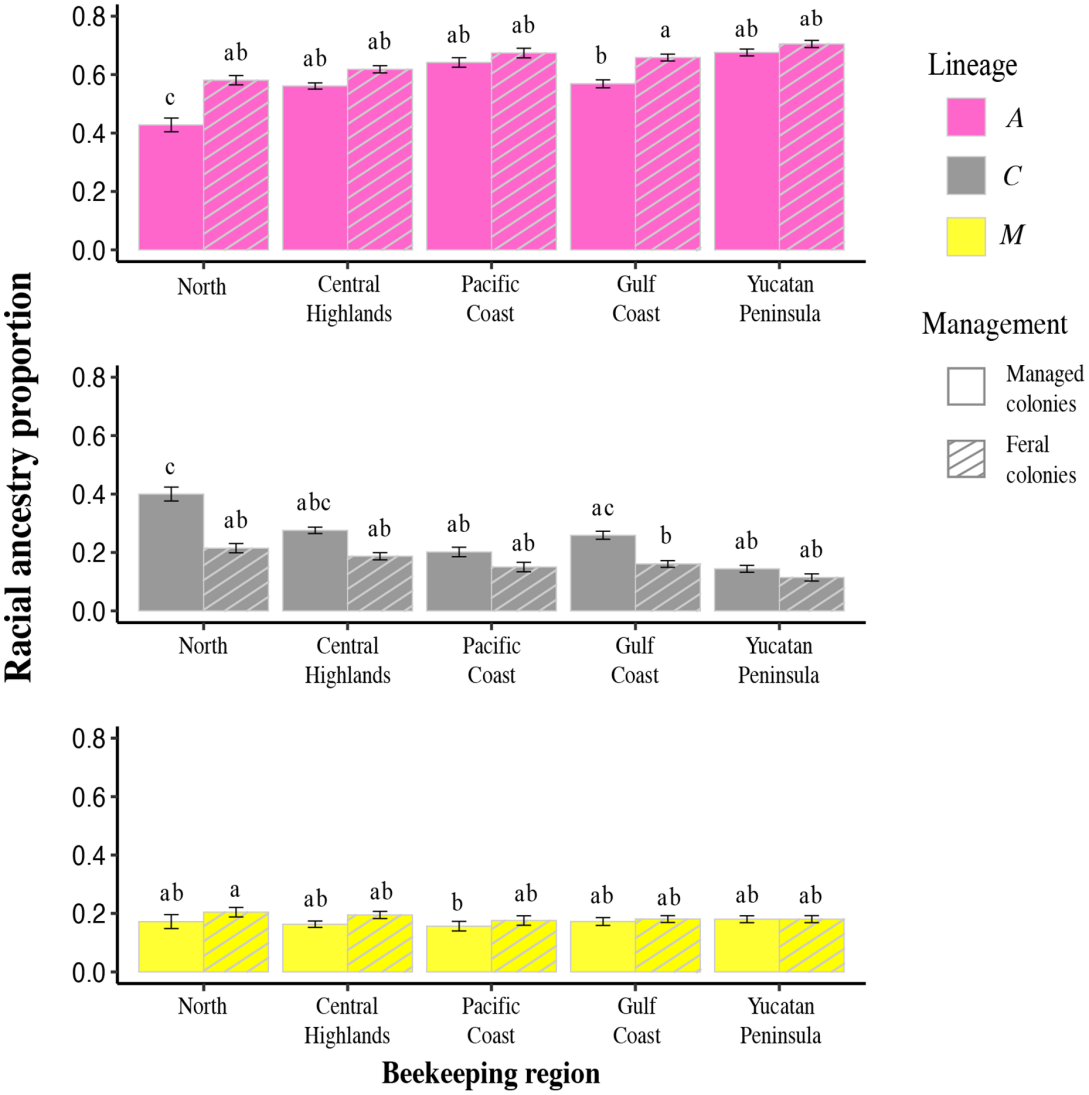
Proportions of nuclear racial ancestry derived from *A* (in pink), *C* (in gray) and *M* (in yellow) lineages in each beekeeping region. Solid bars and dashed bars represent managed and feral colonies, respectively. Different letters above the bars indicate statistically significant differences (*p* < 0.05).

The GLIMMIX analysis showed differences in the proportion of *A* and *C* ancestry between beekeeping regions (*F*_4,59_ = 8.47, *p* < 0.0001; *F*_4,59_ = 5.58, *p* = 0.0007; respectively), as well as management types (*F*_1,432_ = 24.69, *p* < 0.0001; *F*_1,432_ = 26.92, *p* < 0.0001; respectively) and no significant interaction between region and management types (*F*_4,432_ = 1.98, *p* = 0.0962; *F*_4,432_ = 1.54, *p* = 0.1883; respectively). For the proportion of *M* ancestry, differences were observed concerning the management type (*F*_1,432_ = 7.22, *p* = 0.0075), but not for the beekeeping region (*F*_4,59_ = 0.99, *p* = 0.4225), nor for the interaction between region and management type (*F*_4,432_ = 0.78, *p* = 0.5375). Feral honey bee colonies had a higher *A* ancestry (0.61) than managed honey bee colonies (0.57) (*F*_1,432_ = 24.69, *p* < 0.0001), whereas managed honey bee colonies had higher *C* ancestry (0.26) than feral colonies (0.22) (*F*_1,432_ = 26.92, *p* < 0.0001) (Figure 5). The proportion of African ancestry decreased in the managed honey bee colonies from the North and Gulf Coast beekeeping regions, in reciprocity with the increase of the *C* lineage in these honey bee colonies (Figure 5).

The GAMLSS analysis revealed that both managed and feral honey bee colonies had a significant negative relationship between the proportion of African nuclear ancestry with latitude (estimate_managed honey bees_ = −0.0459, SE = 0.0052, *t*‐value_managed honey bees_ = −8.899, *p* = <2e−16; estimate_feral honey bees_ = −0.0261, SE = 0.0059, *t*‐value_feral honey bees_ = −4.443, *p* = 2.1E−0.5) and the mtDNA lineage (estimate_managed honey bees_ = 0.3664, SE = 0.0348, *t*‐value_managed honey bees_ = 10.542, *p* = <2e−16); (estimate_feral honey bees_ = −0.3638, SE = 0.0845, *t*‐value_feral honey bees_ = 4.307, *p* = 3.6E−05), but no relationship was observed with altitude (estimate_managed honey bees_ = −4E−05, SE = 2.6E−05, *t*‐value_managed honey bees_ = −1.573, *p* = 0.1167; estimate_feral honey bees_ = −4E−05, SE = 3.1E−05, *t*‐value_feral honey bees_ = −1.315, *p* = 0.1912).

### 3.3 Comparison of SNPs and COI‐COII analysis

**FIGURE 6:**
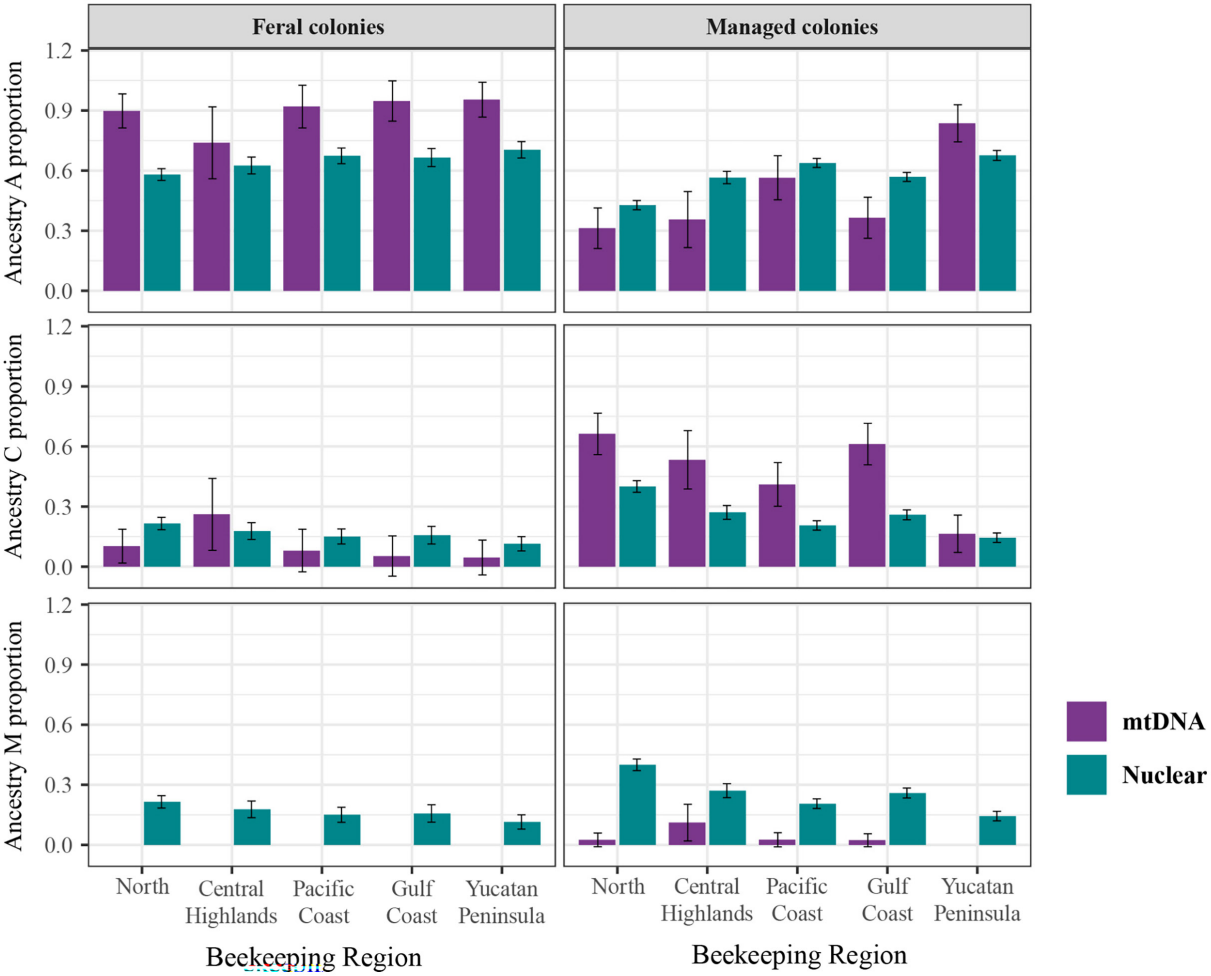
Proportions of African (*A*), Eastern (*C*), and Western European (*M*) derived ancestry in feral and managed honey bee colonies. Purple bars show mtDNA ancestry and blue bars the nuclear ancestry. Error bars represent 95% confidence intervals.

In all beekeeping regions except the Central Highlands, feral honey bee colonies had a significantly higher proportion of African mtDNA ancestry than African nuclear ancestry (Figure 6). In contrast, managed honey bee colonies had a significantly higher proportion of African nuclear ancestry than African mtDNA ancestry in the Central Highlands and the Gulf Coast beekeeping regions, except those of the Yucatan Peninsula, where the proportion of African mtDNA ancestry was significantly higher than African nuclear ancestry. In the North and Pacific Coast regions, there was a trend toward a higher proportion of African nuclear ancestry than African mtDNA ancestry, but it was not significantly different (Figure 6). Feral honey bee colonies in all beekeeping regions, except the Central Highlands, had a significantly higher proportion of nuclear than mtDNA ancestry from Eastern Europe. The Central Highlands showed a higher proportion of mtDNA ancestry than nuclear ancestry from Eastern Europe, but it was not significantly different (Figure 6). Managed colonies in all beekeeping regions, except the Yucatan Peninsula, had significantly higher mtDNA than nuclear proportions of Eastern European ancestry. The Yucatan Peninsula showed the same trend but was not significantly different (Figure 6). Finally, managed and feral honey bee colonies in the five beekeeping regions had a higher nuclear than mtDNA proportion of Western European ancestry. Interestingly, Western European mtDNA ancestry is not present in feral honey bee colonies from any beekeeping region or in managed honey bee colonies from the Yucatan Peninsula.

## 4 DISCUSSION

In this study, we provide a detailed analysis of racial admixture in managed and feral colonies of *A. mellifera* from Mexico, using sequences of the intergenic mitochondrial region COI‐COII and 94 ancestrally informative nuclear SNPs. Our analysis, incorporating mitochondrial sequences and nuclear SNP data, reveals the prevalence of Africanized honey bee hybrids, with a higher proportion of the *A* lineage, in the Mexican honey bee populations.

Analysis of the mitochondrial COI‐COII intergenic region revealed a high haplotype diversity, principally in the African lineage, where 65% of the haplotypes documented are novel variants. This great diversity is similar to that found in other neotropical countries, such as Colombia and Peru (17 and 16 African haplotypes in total, respectively) (Chávez‐Galarza et al., 2021; Tibatá et al., 2018). This result suggests that the genetic diversity of Africanized honey bees has not been lost through genetic drift during their spread, but rather, it appears to have been maintained and probably expanded as a result of recent mutations. This result is similar to other studies that have analyzed the haplotype diversity of *A. mellifera* (Alburaki et al., 2023; Hunde et al., 2023; Zhao et al., 2014). The most frequent African haplotypes recorded in our analysis were variants of haplotypes A1 and A4, with a predominance of A1 (Figure 3c). While the A4 haplotypes are characteristic of South African honey bees, the A1 haplotype has been reported in bees from Africa (*A. m. scutellata*) and the Iberian Peninsula (*A. m. iberiensis* E.) (Chávez‐Galarza et al., 2017; Franck et al., 1998; Garnery et al., 1993, 1995; Techer et al., 2017). Therefore, it has been proposed that the origin of the A1 haplotypes in the Americas could be either from the Iberian Peninsula or via the introduction of *A. m. scutellata* (Clarke et al., 2001; Collet et al., 2006). Collet et al. (2006) suggested a South African origin (via *A. m. scutellata*) of the A1 haplotypes based on the low frequency of A1 haplotypes on the continent before the expansion of Africanized swarms and its increase after the Africanization process. They also proposed an African origin if the A1 haplotypes in the southern part of the continent and those in North America are the same. Our data support this proposal since the variants of the A1 haplotype recorded in Mexico are the same as those found in South and North America. For example, the A1e haplotype, found in high frequency in the five beekeeping regions of Mexico (e.g., A1e = 35.2% of 495 colonies analyzed), is also common in the south of the continent, in Colombia (31.9% of 645 analyzed colonies) (Tibatá et al., 2018), and in the south of the USA (Alburaki et al., 2023). Another point that supports the African origin is that the variant A1e has been found in Africa (Amakpe et al., 2018; Coulibaly et al., 2019) but not in the Iberian Peninsula despite being one of the most sampled regions (Cánovas et al., 2008; Chávez‐Galarza et al., 2017; Henriques et al., 2022; Pinto et al., 2012, 2013). In contrast, the haplotype diversity in the *C* and *M* lineages was comparatively lower than in the *A* lineage, and no novel haplotypes of the *C* lineage were found in our study. The most frequent *C* haplotypes were C1 and C2j, which have been associated with the subspecies *A. m. ligustica* S. and *A. m. carnica* P., respectively (Muñoz et al., 2009; Özdil et al., 2022). In our samples, these haplotypes were mainly recorded in managed colonies throughout Mexico. Similarly, in Peru and USA, these haplotypes have been found in high frequencies in managed honey bee colonies (Alburaki et al., 2023; Chávez‐Galarza et al., 2021). Their high frequency is likely due to beekeepers selecting these haplotypes for their desirable phenotypic traits such as docility and productivity (Delaney et al., 2009; Franck et al., 2000; Magnus et al., 2011; Schiff & Sheppard, 1996).

The nuclear analysis revealed that Mexican honey bee colonies had principally African ancestry and a minor proportion of European racial ancestry, with admixture patterns varying across the country, according to management, beekeeping regions, and latitude. Geographic variation of nuclear admixture was characterized by an increase of the *A* ancestry (with a consequent decrease of *C* ancestry) in lower latitudes. These results are in line with previous studies that found a latitudinal effect on African ancestry (Agra et al., 2018; Porrini et al., 2022; Sheppard et al., 1991; Whitfield et al., 2006; Zárate et al., 2022), suggesting that *A* lineage may be disadvantaged at higher latitudes and altitudes due to lower temperatures and longer winters (Calfee et al., 2020; Harrison et al., 2006). In managed colonies of the Northern beekeeping region, there was a higher decline in the proportion of African nuclear ancestry compared to feral colonies. This result could be explained by recurrent introduction of European honey bee queens in this region. In the feral honey bee colonies, unlike the managed honey bee colonies, the *C* and *M* ancestry proportions were low (<22%), and no differences were observed between the beekeeping regions (Figure 5).

The low proportion of *M* ancestry in honey bee colonies in Mexico is similar to those found in other countries in the Americas (Harpur et al., 2020; Nelson et al., 2017). These previous studies have found that although the *M* lineage is not used currently in commercial beekeeping (Kraus et al., 2007), Africanized honey bee colonies always have a proportion of this lineage in their genome, which has been associated with the introgression of favorable alleles of *M* lineage into the Africanized honey bee genome (Cridland et al., 2018; Whitfield et al., 2006; Zárate et al., 2022; Zayed & Whitfield, 2008). Nelson et al. (2017), using a genome‐wide analysis of admixture and adaptation, documented that favorable alleles of *M* ancestry, related to reproductive and foraging traits, have introgressed into the Africanized honey bee genome and have been favored by natural selection. Unlike honey bee colonies in Mexico, Brazilian honey bee colonies exhibit a small proportion of *C* ancestry, so it would be interesting to determine if the same regions of the genome under selection are also found in Mexican populations.

The results showed a significant positive relationship between the African maternal haplotypes and the proportion of African nuclear ancestry (Table 2), suggesting that most colonies in Mexico result from queen bees with African mtDNA mating with drones with predominantly African nuclear ancestry. However, this pattern varies in terms of the management conditions and the beekeeping region. A higher proportion of African mtDNA ancestry (with the almost disappearance of European mtDNA) than African nuclear ancestry was recorded in the managed honey bee colonies from the Yucatan Peninsula and all feral honey bee colonies, except for the Central Highlands. The low European mtDNA frequencies found in feral colonies of Mexico are similar to those observed in other feral Africanized honey bee populations (Lobo, 1995; Sheppard et al., 1991). In feral populations, the disappearance of the European mtDNA is likely due to an extensive mating of European honey bee queens with Africanized honey bee drones after the arrival of the Africanized honey bee, followed by a pattern of queen development that favors hybrids with African mtDNA compared to their counterparts with European mtDNA. Matings of F1 queens with maternal European ancestry with African honey bee drones probably resulted in colonies with low fitness and the eventual loss of European mtDNA from feral populations (Lobo, 1995; Taylor, 2009). Additionally, our study showed that African mtDNA may have spread as a continuous lineage in feral colonies, with little contribution from European lineages, which are less prone to swarm and produce queen bees (Hall & Muralidharan, 1989; Smith et al., 1989). In the managed colonies of the Yucatan Peninsula, the higher proportion of African mtDNA ancestry than African nuclear ancestry is likely due to the management practices of beekeepers that have reduced to a minimum the requeening (personal communication with beekeepers) or an intense incorporation of feral colonies into apiaries (Quezada‐Euán et al., 1996). Domínguez‐Ayala et al. (2016) found a minor proportion of honey bee colonies with mtDNA of African origin in the same beekeeping region. Recently, in 2022, a program to requeen colonies with presumably European stock brought from other regions of Mexico has taken place in Yucatan (https://www.yucatan.gob.mx/saladeprensa/ver_nota.php?id=6514). At least 3200 queens have been delivered to apiaries. This honey bee stock has not been genotyped, and due to the prevalence of African ancestry across Mexico, the increase in the proportion of African mtDNA ancestry in this region would likely have occurred through these programs.

**TABLE 2:** Results of the GAMLSS Models showing the relationship between altitude, latitude, and lineage (African or European, based on mtDNA) with the nuclear African proportion (based on ancestry informative SNPs) in managed and feral honey bee colonies.

Management	Independent variable	Estimate	SE	*t* value	Pr(>|*t*|)
Managed colonies	Intercept	−1.848	0.7356	−2.512	0.0125*
	Altitude	−0.0000408	0.0000259	−1.573	0.1167
	Latitude	−0.04585	0.005153	−8.899	<2e−16***
	Lineage (African)	0.3664	0.03476	10.542	<2e−16***
Feral colonies	Intercept	−1.889	0.8925	−2.116	0.0365*
	Altitude	−0.00004031	0.00003065	−1.315	0.1912
	Latitude	−0.02614	0.005884	−4.443	0.000021***
	Lineage (African)	0.3638	0.08447	4.307	0.0000357***

Asterisks indicate level of statistical significance: *p ≤ 0.05, **p ≤ 0.01, ***p ≤ 0.001.

Contrary to feral colonies of the five beekeeping regions and the managed honey bee colonies of the Yucatan Peninsula, managed colonies in the Central Highlands and Gulf Coast beekeeping regions showed a lower proportion of African mtDNA ancestry and a higher proportion of African nuclear ancestry. Interestingly, although European mtDNA ancestry is more frequent in these colonies, the proportion of African nuclear ancestry remains consistent across the five beekeeping regions, except the North region. A higher reintroduction of European honey bee queens (principally *C* lineage) may have reduced the proportion of African mtDNA ancestry. However, the mating of these introduced European honey bee queens with African drones, which are highly predominant in drone congregation areas (Collet et al., 2009; Galindo‐Cardona et al., 2020; Rinderer et al., 1987), may maintain the African nuclear ancestry in these colonies. The higher proportion of African nuclear ancestry may be exacerbated in areas with a high density of feral Africanized colonies, such as regions with natural or semi‐natural areas with more availability of floral resources and nesting sites (Balbino‐Olvera et al., 2024). However, studies analyzing the honey bee colonies' abundance and racial ancestry in natural habitat conditions are scarce.

The Africanization of honey bees was considered one of the main problems affecting beekeeping in Mexico (Guzmán‐Novoa et al., 2011) and to prevent the initial predictions of a collapse of Mexico's beekeeping, a management program (National Program for the Control of the African Bee) was initiated before its arrival. After almost 40 years, this program is still in place today under the supervision and regulation of the Ministry of Agriculture and Rural Development (SADER) and the officially approved policy NOM‐002‐SAG/GAN‐2016. A recent study using long‐term beekeeping data in Mexico reveals that although there was a decrease in total production of honey in the 80s—likely related to the Africanization process—in the last years, honey production has partially rebounded and remained relatively stable (Balvino‐Olvera et al., 2023). Balvino‐Olvera et al. (2023) documented a notable increase in the percentage change in the number of hives (0.01–0.31%) in tropical areas, which according to our results, have predominantly African ancestry. In contrast, northern regions of the country (with more European ancestry according to our results) saw declining trends in honey bee colonies (Balvino‐Olvera et al., 2023). It has been proposed that genetic improvement programs using introduced European honey bee queens can increase European allele frequencies and honey production (Guzman‐Novoa et al., 2020; Guzmán‐Novoa & Page Jr., 1999). However, our data reveal that, in the long term, these programs may not be viable due to feral populations being mostly Africanized. Thus, the constant mating of European honey bee queens with Africanized drones in DCAs favors a constant gene flow of Africanized alleles into managed apiaries. Besides, Africanized honey bees have useful attributes for beekeeping, such as resistance against the parasitic mite—*Varroa destructor*—to viruses and climate change (Guichard et al., 2020; Medina et al., 2018; Ramos‐Cuellar et al., 2022). Given the adaptability to a wide range of environments and the beneficial attributes of the Africanized honey bee, the use of these honey bees should represent a profitable option to improve beekeeping in Mexico. For example, in the Yucatan Peninsula, Africanized honey bee selection has resulted in higher honey yields, disease resistance, and lower defensive capacity (Quezada‐Euán et al., 2008).

Our results show that Mexican honey bees are mainly African‐derived and that the European mitochondrial lineage is present due to the recurrent introduction of European honey bee queens in managed colonies or better adaptation of lineages with maternal European ancestry in some regions. The introduction of European honey bee queens in Mexico is mainly because the “National Program for the Control of the African Bee” mandates that beekeepers must change the queen bees of each hive at least once a year, replacing them with others of European origin or genetically improved (NOM‐002‐SAG/GAN‐2016). This Official Program also mandates that the movement of hives, queen bees, bee nuclei, or beekeeping biological material from Africanized honey bee control areas to Africanized honey bee‐free areas will not be permitted (NOM‐002‐SAG/GAN‐2016). Our study shows a divorce between the biological reality 30 years after the migration of Africanized honey bees from Brazil into Mexico and the current “Mexican Official Government Program for the Control of the African Bee”. Our study demonstrates mainly African nuclear and mitochondrial ancestry for managed and nearby feral honey bee colonies in Mexico. Therefore, there is a need to incorporate science into policy in this Mexican Official Program or other equivalent programs in other countries.

## 5 CONCLUSION

Our study demonstrates that Africanized honey bee populations are spread and established along Mexico. The proportion of African (both maternal and nuclear) ancestry is higher in feral colonies and beekeeping regions of lower latitudes. Mitochondrial DNA marker indicates that, although European maternal lineages have almost disappeared from feral populations, they are maintained in managed populations by beekeepers and queen breeders through the introduction of European honey bee queens, predominantly in regions with highlands (Central highlands) and higher latitudes (North) and possibly natural selection for European traits in these regions. However, the high proportion of African nuclear ancestry in managed honey bee colonies indicates that these introduced European honey bee queens (with European mtDNA) mate frequently with Africanized drones. This result highlights the limitations of management and government programs to contain the Africanization honey bee process. Our results suggest that after 30 years of the arrival of Africanized honey bees to Mexico, these Africanized hybrids are genetically diverse and well established in all beekeeping regions of Mexico. Therefore, this lineage is well adapted to highly variable biophysical regions and should be considered in any breeding program for sustainable beekeeping.

## FUNDING INFORMATION

This study was supported by grants from Universidad Nacional Autónoma de México (PAPIIT IV200418, IN224920, IN219021, IN225924), SADER‐CONAHCyT 291,333, CONAHCyT‐National Repositories 271,432, CONAHCyT‐UNAM‐UAGro‐UMSH to Laboratorio Nacional de Análisis y Síntesis Ecológica LANASE (2015LN250996, 2016‐LN271449, 2017‐LN280505, 2018‐LN293701, 2019‐LN299033, 2020‐LN314852, 2021‐LN 315810), LANASE‐CIC‐UNAM 2015–2024, and Programa Iberoamericano de Ciencia y Tecnología para el Desarrollo RED CYTED‐SEPODI (417RT0527).

## CONFLICT OF INTEREST STATEMENT

The authors declare no conflict of interest.

## Supporting information


Appendix S1.

## Data Availability

The data that support the findings of this study are openly available in the NCBI Sequence Read Archive (Genbank accessions PP390502‐PP390532) and the Dryad repository at doi: 10.5061/dryad.0p2ngf28j.
