# Supplementary material for: Dominance of African racial ancestry in honey bee colonies of Mexico 30 years after the migration of hybrids from South America
Source: Evol Appl. 2024 Jun 24;17(6):e13738. doi: 10.1111/eva.13738 (PMC11196837; doi:10.1111/eva.13738)
Supplement: Supplementary file 1 — Appendix S1. [file EVA-17-e13738-s001.docx]

**Supporting Information**

**Figure S1**. Relationship between allele frequency estimates from pooling and individual genotyping of five honey bee colonies (r = 0.999). Pooled DNA samples consist of 20 worker bees per colony. Allele frequencies were calculated using ten ancestry informative SNPs. Different colors and symbols represent different primers and hives, respectively.


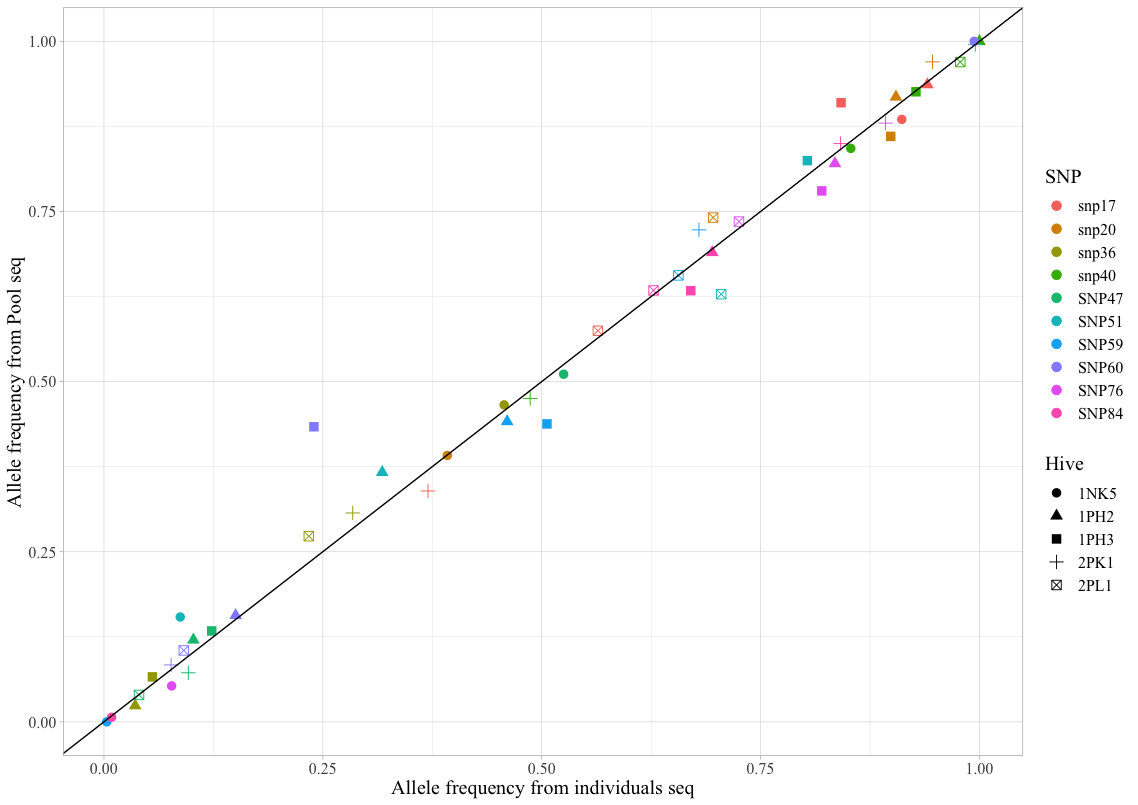


**Figure S2**. Ternary plot by beekeeping region showing the racial admixture of the three main ancestral lineages of honey bees in Mexico (A, C and M). Managed honey bee colonies are represented by circles and wild honey bee colonies by squared plus sign.

**
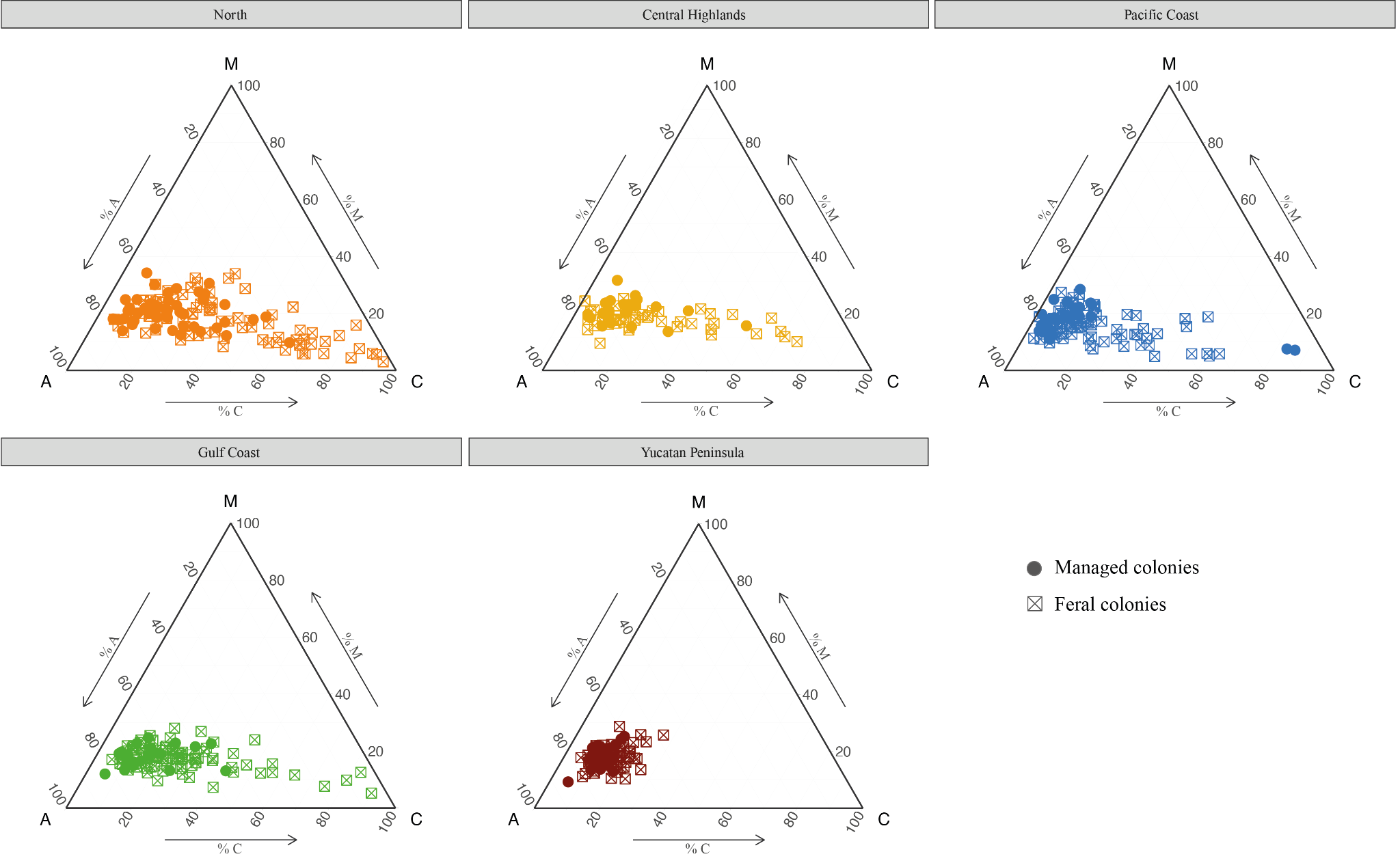
**

**Table S1.** Number of feral and managed honeybee colonies collected per beekeeping regions of Mexico.

| Beekeeping  region | Site | N colonies | | |
| --- | --- | --- | --- | --- |
|  |  | Managed | Feral | Total |
| North | 1) BCNLer | 5 | 8 | 13 |
|  | 2) BCNEns | 5 | 2 | 7 |
|  | 3) BCSCab | 6 | - | 6 |
|  | 4) BCSPed | 5 | 5 | 10 |
|  | 5) BCSPit | 6 | 5 | 11 |
|  | 6) BCSViz | - | 7 | 7 |
|  | 7) ChiJim | 5 | - | 5 |
|  | 8) ChiParral | - | 4 | 4 |
|  | 9) Coa4C | - | 4 | 4 |
|  | 10) CoahSB | 6 | 1 | 7 |
|  | 11) DgoILR | 5 | 5 | 10 |
|  | 12) DgoRC | 6 | 2 | 8 |
|  | 13) DgoSC | 11 | 6 | 17 |
|  | 14) NLSab | 12 | - | 12 |
|  | 15) SLPMat | 3 | - | 3 |
|  | 16) SonHer | 5 | 1 | 6 |
|  | **Total** | **80** | **50** | **130** |
| Central highlands | 1) AgsHer | 4 | 5 | 9 |
|  | 2) AgsJM | 4 | 5 | 9 |
|  | 3) CuePaz | - | 4 | 4 |
|  | 4) GroTax | 3 | - | 3 |
|  | 5) GtoCap | - | 5 | 5 |
|  | 6) JalJamay | 4 | 1 | 5 |
|  | 7) JalPrim | 8 | 4 | 12 |
|  | 8) JalProv | 4 | - | 4 |
|  | 9) MichFri | 6 | - | 6 |
|  | 10) MichPatz | 6 | - | 6 |
|  | 11) MichSJT | 4 | - | 4 |
|  | 12) MichTri | 6 | 3 | 9 |
|  | **Total** | **49** | **27** | **76** |
| Pacific coast | 1) ChpsCa | 9 | 1 | 10 |
|  | 2) ChpsCo | 5 | 2 | 7 |
|  | 3) ColPoz | 7 | - | 7 |
|  | 4) ColSFer | 9 | - | 9 |
|  | 5) GroJobI | 9 | - | 9 |
|  | 6) GroJobII | 8 | - | 8 |
|  | 7) GroPor | - | 4 | 4 |
|  | 8) GroTet | 11 | - | 11 |
|  | 9) GroUAGro | 6 | - | 6 |
|  | 10) JalCha | - | 5 | 5 |
|  | 11) OaxBCh | 4 | - | 4 |
|  | 12) SinBot | 5 | 4 | 9 |
|  | 13) SinFue | 5 | 5 | 10 |
|  | 14) TepAgu | 5 | 4 | 9 |
|  | **Total** | **83** | **25** | **108** |
| Gulf coast | 1) SLPCent | 11 | 1 | 12 |
|  | 2) SLPSAnt | 9 | 1 | 10 |
|  | 3) TabMarIn | 11 | - | 11 |
|  | 4) TabMcha | 5 | 2 | 7 |
|  | 5) TampsCar | 5 | - | 5 |
|  | 6) TampsCarM | 4 | - | 4 |
|  | 7) TampsMor | - | 3 | 3 |
|  | 8) TampsTN | 11 | 5 | 16 |
|  | 9) VerLl | 5 | - | 5 |
|  | 10) VerSTux | 10 | 5 | 15 |
|  | 11) VerTemap | 9 | - | 9 |
|  | 12) VerTeo | 9 | 4 | 13 |
|  | **Total** | **89** | **21** | **110** |
| Yucatan Peninsula | 1) CampCC | 8 | 3 | 11 |
|  | 2) CampPotr | 5 | - | 5 |
|  | 3) CampRA | 10 | - | 10 |
|  | 4) CampXpuj | 9 | 4 | 13 |
|  | 5) QRooAO | - | 11 | 11 |
|  | 6) QRooLech | 9 | 1 | 10 |
|  | 7) YucGall | 8 | - | 8 |
|  | 8) YucKab | - | 4 | 4 |
|  | 9) YucSJTzal | 7 | - | 7 |
|  | 10)YucTekax | 6 | - | 6 |
|  | **Total** | **62** | **23** | **85** |
| **Total** |  | **363** | **146** | **509** |

**Table S2.**  Lineages, sequences, fragment sizes, and accession numbers in the GeneBank of haplotypes obtained from feral and managed honey bee colonies in Mexico.

| Haplotype | Lineage | Sequence | DraI fragment sizes (bp) | Total fragment size (bp) | Accession number |
| --- | --- | --- | --- | --- | --- |
| A1e | A | P0Q | 47/108/483 | 638 | PP390502 |
| A1v | A | P0Q | 47/108/483 | 638 | PP390503 |
| A1w | A | P0Q | 47/109/483 | 639 | PP390504 |
| A1x | A | P0Q | 47/108/482 | 637 | PP390505 |
| A4aa | A | P0QQ | 47/107/192/482 | 828 | PP390506 |
| A4ab | A | P0QQ | 47/108/192/487 | 834 | PP390507 |
| A4ac | A | P0QQ | 47/108/192/480 | 827 | PP390508 |
| A4ad | A | P0QQ | 47/109/193/483 | 832 | PP390509 |
| A4c' | A | P0QQQ | 47/107/192/192/482 | 1020 | PP390510 |
| A4p | A | P0QQ | 47/109/192/483 | 831 | PP390511 |
| A4t | A | P0QQ | 47/108/192/483 | 830 | PP390512 |
| A4x | A | P0QQ | 47/109/192/482 | 830 | PP390513 |
| A4y | A | P0QQ | 47/108/192/482 | 829 | PP390514 |
| A4z | A | P0QQ | 47/107/192/483 | 829 | PP390515 |
| A16' | A | P1QQQ | 47/93/867 | 1007 | PP390516 |
| A16m' | A | P1QQQ | 47/93/866 | 1006 | PP390517 |
| A16n' | A | P1QQQ | 47/94/866 | 1007 | PP390518 |
| A26e | A | P0QQ | 47/96/192/481 | 816 | PP390519 |
| C1 | C | Q | 47/41/64/420 | 572 | PP390520 |
| C2 | C | Q | 47/40/64/420 | 571 | PP390521 |
| C2c | C | Q | 47/40/64/420 | 571 | PP390522 |
| C2j | C | Q | 47/40/64/420 | 571 | PP390523 |
| C2l | C | Q | 47/40/64/419 | 570 | PP390524 |
| C2s | C | Q | 47/40/63/421 | 571 | PP390525 |
| C3 | C | Q | 47/40/63/420 | 570 | PP390526 |
| M7 | M | PQQ | 47/95/65/131/65/422 | 825 | PP390527 |
| M7b' | M | PQQQ | 47/95/65/131/65/131/65/420 | 1019 | PP390528 |
| M7c' | M | PQQQ | 47/95/65/131/65/131/65/422 | 1021 | PP390529 |
| M7p | M | PQQ | 47/95/65/131/65/421 | 824 | PP390530 |
| O6 | O | P0QQ | 47/107/67/129/67/419 | 836 | PP390531 |
| O6a | O | P0QQ | 47/107/67/129/67/420 | 837 | PP390532 |

**Table S3.** Haplotypes recorded in feral (F) and managed (M) honeybee colonies in the five beekeeping regions of Mexico.

| **Lineage** | | **Haplotype** | **North** | | **Central highlands** | | **Pacific coast** | | **Gulf coast** | | **Yucatan Peninsula** | | **Total** | **%** |
| --- | --- | --- | --- | --- | --- | --- | --- | --- | --- | --- | --- | --- | --- | --- |
|  | Sublineage |  | F | M | F | M | F | M | F | M | F | M |  |  |
| A | AI | A1e | 35 | 14 | 8 | 4 | 13 | 29 | 9 | 14 | 14 | 34 | 174 | 35.2 |
|  |  | A1v | 5 | 7 | 2 | 4 | 4 | 4 | 1 | 5 | 2 | 8 | 42 | 8.5 |
|  |  | A1w | 0 | 1 | 0 | 0 | 0 | 0 | 0 | 0 | 0 | 0 | 1 | 0.2 |
|  |  | A1x | 0 | 0 | 1 | 0 | 1 | 1 | 0 | 0 | 0 | 1 | 4 | 0.8 |
|  |  | A4p | 1 | 2 | 3 | 5 | 2 | 4 | 1 | 6 | 0 | 1 | 25 | 5.1 |
|  |  | A4t | 2 | 0 | 0 | 0 | 0 | 0 | 2 | 0 | 0 | 0 | 4 | 0.8 |
|  |  | A4x | 0 | 0 | 1 | 2 | 3 | 5 | 1 | 3 | 0 | 1 | 16 | 3.2 |
|  |  | A4y | 1 | 0 | 0 | 0 | 0 | 0 | 1 | 0 | 1 | 0 | 3 | 0.6 |
|  |  | A4z | 0 | 0 | 1 | 0 | 0 | 0 | 0 | 0 | 1 | 0 | 2 | 0.4 |
|  |  | A4aa | 0 | 0 | 0 | 0 | 0 | 0 | 0 | 1 | 0 | 0 | 1 | 0.2 |
|  |  | A4ab | 0 | 0 | 0 | 0 | 0 | 0 | 0 | 1 | 0 | 0 | 1 | 0.2 |
|  |  | A4ac | 0 | 0 | 0 | 0 | 0 | 0 | 1 | 0 | 0 | 0 | 1 | 0.2 |
|  |  | A4ad | 0 | 0 | 0 | 0 | 0 | 0 | 0 | 0 | 0 | 1 | 1 | 0.2 |
|  |  | A4c' | 0 | 0 | 0 | 0 | 0 | 0 | 0 | 1 | 0 | 0 | 1 | 0.2 |
|  |  | A26e | 0 | 0 | 0 | 0 | 0 | 0 | 0 | 0 | 0 | 1 | 1 | 0.2 |
|  | AIII | A16' | 0 | 0 | 0 | 0 | 0 | 0 | 1 | 0 | 1 | 0 | 2 | 0.4 |
|  |  | A16m' | 0 | 1 | 2 | 1 | 0 | 0 | 1 | 0 | 1 | 1 | 7 | 1.4 |
|  |  | A16n' | 0 | 0 | 0 | 0 | 0 | 1 | 0 | 0 | 0 | 0 | 1 | 0.2 |
| C | | C1 | 3 | 27 | 4 | 11 | 1 | 11 | 0 | 36 | 0 | 0 | 93 | 18.8 |
|  |  | C2 | 0 | 3 | 1 | 1 | 0 | 0 | 0 | 5 | 0 | 0 | 10 | 2.0 |
|  |  | C2c | 0 | 4 | 0 | 0 | 0 | 9 | 0 | 4 | 0 | 0 | 17 | 3.4 |
|  |  | C2j | 2 | 17 | 2 | 13 | 1 | 10 | 0 | 9 | 1 | 10 | 65 | 13.1 |
|  |  | C2l | 0 | 0 | 0 | 0 | 0 | 0 | 1 | 2 | 0 | 0 | 3 | 0.6 |
|  |  | C2s | 0 | 2 | 0 | 0 | 0 | 0 | 0 | 0 | 0 | 0 | 2 | 0.4 |
|  |  | C3 | 0 | 0 | 0 | 0 | 0 | 2 | 0 | 0 | 0 | 0 | 2 | 0.4 |
| M | | m7 | 0 | 0 | 0 | 0 | 0 | 1 | 0 | 0 | 0 | 0 | 1 | 0.2 |
|  |  | M7b' | 0 | 2 | 0 | 4 | 0 | 0 | 0 | 1 | 0 | 0 | 7 | 1.4 |
|  |  | M7c' | 0 | 0 | 0 | 1 | 0 | 0 | 0 | 1 | 0 | 0 | 2 | 0.4 |
|  |  | M7p | 0 | 0 | 0 | 0 | 0 | 1 | 0 | 0 | 0 | 0 | 1 | 0.2 |
| O | | O6 | 0 | 0 | 0 | 0 | 0 | 0 | 0 | 0 | 1 | 3 | 4 | 0.8 |
|  |  | O6a | 0 | 0 | 0 | 0 | 0 | 0 | 0 | 0 | 0 | 1 | 1 | 0.2 |
|  |  | **Total** | **49** | **80** | **25** | **46** | **25** | **78** | **19** | **89** | **22** | **62** | **495** | **100** |
